# Isolation and characterization of acetoin-producing *Bacillus* species from *Salicornia europaea* rhizosphere with evaluation of their saline-alkaline adaptation

**DOI:** 10.3934/microbiol.2026012

**Published:** 2026-05-12

**Authors:** Yao Li, Hanwen Cui, Enbiao Wang, Tianwen Cao, Yao Meng, Mai Du, Tingting Li, Nan Gao

**Affiliations:** 1 School of Biotechnology and Pharmaceutical Engineering, Nanjing Tech University, Nanjing 211816, China; 2 Shenyang Research Institute of Chemical Industry, Shenyang 110021, China

**Keywords:** *Salicornia* sp., acetoin-producing bacteria, *Bacillus*, PGPR, saline‑alkali soil

## Abstract

Soil salinization is a major constraint on global agricultural productivity, and the application of plant growth‑promoting rhizobacteria offers an important solution for the management and utilization of saline-alkali land. In this study, we aimed to isolate and characterize beneficial rhizobacteria from the halophyte *Salicornia europaea* growing in coastal saline‑alkali soils, and to evaluate their potential in promoting plant growth under stressful conditions. Among 57 purified bacterial isolates, 3 acetoin‑producing strains, identified as *Bacillus* sp. HPZ‑9, HPZ‑47, and HPZ‑50, were selected. They produced acetoin at concentrations of 2.30 g·L^−1^, 2.73 g·L^−1^, and 2.77 g·L^−1^, respectively. All strains exhibited broad environmental adaptability, sustaining growth and acetoin production across a pH range of 5–11, NaCl concentrations of 0.3–1.2 mol·L^−1^, and under moderate drought stress simulated with 20% polyethylene glycol 6000. Notably, HPZ‑9 tolerated NaCl levels up to 2.4 mol·L^−1^. In pot experiments with maize seedlings, inoculation with these strains significantly increased plant height, stem diameter, leaf length, and fresh and dry biomass of shoots and roots compared to the uninoculated controls (*p* < 0.05). The most notable improvement was observed in dry biomass, which increased by 49% to 60%. In conclusion, the selected *Bacillus* strains show strong plant growth‑promoting traits and high tolerance to saline‑alkaline stress. These findings highlight their potential as effective microbial inoculants for promoting crop health and improving saline-alkali soils.

## 1. Introduction

Soil salinization is a critical ecological issue limiting global agricultural sustainability, leading to cropland degradation, reduced crop yields, and ecological environment deterioration. While physical and chemical remediation techniques offer certain benefits, their high costs and potential for secondary pollution limit their applications. Consequently, environmentally friendly and sustainable biological remediation strategies have garnered significant attention. Among these, the application of plant growth-promoting rhizobacteria (PGPR) is particularly promising. PGPR are rhizosphere-colonizing bacteria that directly or indirectly promote plant growth, suppress plant pathogens, and enhance plant resilience to environmental stresses [1]. PGPR facilitate plant growth through direct mechanisms such as biological nitrogen fixation, phosphate solubilization, and the synthesis of phytohormones [2]. These activities improve nutrient availability and stimulate root development. For instance, application of *Bacillus subtilis* MBI600 and *Bacillus amyloliquefaciens* QST713 significantly increase shoot height and root length in tomato plants [3]. Furthermore, PGPR employ indirect mechanisms, such as the production of hormone and volatile organic compounds (VOCs) to enhance plant stress tolerance [4]–[7].

VOCs are key mediators of plant-microbe interactions. For example, VOCs from *Stutzerimonas stutzeri* NRCB010 significantly enhance biofilm formation, swarming motility, root colonization, and tomato seedling growth under sterile and greenhouse conditions [8]. Similarly, VOCs from bacteria such as *Bacillus licheniformis* and *Bacillus velezensis* have been shown to promote growth in greenhouse pot experiments [9]. Moreover, systematic evaluations indicate that VOCs from certain PGPR strains can modulate plant hormone pathways and positively shape the rhizosphere microbiome, thereby synergistically promoting plant growth [10]. Research on *Bacillus* VOCs confirmed their role in activating plant antioxidant systems and upregulating related gene expression in tomato plants [11]. Among bacterial VOCs, acetoin (3-hydroxy-2-butanone) is a well-characterized bioactive compound. It is widely utilized in the food, chemical, pharmaceutical, and tobacco industries and serves as a secondary metabolite from microorganisms [12]. Initially identified as a plant growth promoter released by *B. subtilis* GB03 [13], acetoin has been consistently shown to enhance biomass in model plants, such as *Arabidopsis thaliana* [14]. Additionally, evidence highlights its broader functional significance, such as enhancing biofilm formation and colonization, and alleviating salt stress in *Sesbania* [15]. Despite these advances, exploring novel PGPR, especially acetoin producers, from extreme habitats like saline-alkaline soils remains crucial for developing targeted inoculants. Halophytes, such as *Salicornia europaea*, are native to coastal saline-alkali areas and possess remarkable phytoremediation capabilities [16]–[18].

Therefore, we aimed to isolate and characterize novel acetoin-producing rhizobacteria from the rhizosphere of *S. europaea*. Our objectives were to: (1) Screen and purify bacterial isolates from this unique niche and preliminarily identify promising acetoin-producing strains; (2) evaluate their growth dynamics, acetoin synthesis, and tolerance to simulated drought, varied pH, and salt stress; and (3) evaluate their growth-promoting potential on maize seedlings through greenhouse pot experiments. The findings are expected to provide valuable germplasm resources for developing specialized microbial inoculants to improve crop resilience and soil health in saline-alkaline ecosystems.

## 2. Materials and methods

### 2.1. Soil sample collection and bacterial isolation

Rhizospheric soil samples were collected on November 14, 2023 from a coastal area (32°46′34″N, 120°56′21″E). Vigorously growing *S. europaea* plants were excavated using a sterile shovel to preserve the root system and adhering soil. The plants with intact rhizosphere soil were placed into sterile sampling bags, sealed after removing surface debris, transported to the laboratory, and stored at −80 °C.

For bacterial isolation, 1 g of soil tightly adhering to the root was suspended in 9 ml of sterile distilled water and vortexed for 30 min. A serial dilution was performed by transferring 1 mL of the suspension into 9 mL of sterile distilled water, repeated consecutively six times [19]. Aliquots (100 µL) from appropriate dilutions were spread-plated onto a modified 1/10 Luria-Bertani (LB) solid medium (1 g·L^−1^ tryptone, 0.5 g·L^−1^ yeast extract, 4.51 g·L^−1^ NaCl, 15 g·L^−1^ agar) to simulate the oligotrophic conditions of saline-alkali soil. Plates were incubated at 30 °C. Distinct colonies were purified via three successive streak plates on the same medium. Pure isolates were preserved at −80 °C for long-term storage.

### 2.2. Strains activation and culture preparation

Preserved strains were retrieved from −80 °C storage, streaked onto 1/10 LB agar plates, and incubated at 30 °C for 24–48 h for activation. A single colony was inoculated into 100 mL of LB liquid medium (10 g·L^−1^ tryptone, 5g·L^−1^ yeast extract, 10 g·L^−1^ NaCl) and incubated as a seed culture at 30 °C with shaking at 200 rpm for 24 h. For fermentation, this seed culture was inoculated at 1% (v/v) into fresh glucose-peptone water (GP) medium (5 g·L^−1^ glucose, 7 g·L^−1^ tryptone, 5 g·L^−1^ K_2_HPO_4_) and incubated under the same conditions for 72 h.

### 2.3. Screening and quantification of acetoin-producing strains

The Voges Proskauer (VP) test was used for preliminary screening of acetoin-producing strains [20]. The VP-positive strains were further used to determine the acetoin concentration [21]. Acetoin concentration in the 72-h fermentation broth was quantified using the creatine colorimetric method [22]. A total 5 mL aliquots of the cultures were taken, centrifuged for 10 min at 4300 × g, and 0.5 mL of the supernatant were mixed with 1 mL of 10% NaOH, 1 mL of 0.5% creatine, and 1 mL of 5% naphthol. The mixture was incubated at 30 °C for 30 min, and the absorbance at 540 nm was measured using a spectrophotometer. Acetoin concentrations were determined using an acetoin standard curve from 0.01 to 1 g·L^−1^. The acetoin-producing rhizobacterial isolate biomass was evaluated by optimal density (OD_540_). Each bacterial culture experiment was conducted in triplicate.

### 2.4. Screening for 1-aminocyclopropane-1-carboxylate deaminase activity

The isolates were assessed for ACC deaminase activity by comparing their growth in Dworkin and Foster (DF) minimal medium (Yuanye Bio-Technology Co., Ltd., Shanghai, China) with and without 1-aminocyclopropane-1-carboxylate (ACC) as the sole nitrogen source. Strains were inoculated into DF and ACC-supplemented DF (ADF) media. Growth was monitored by measuring the optical density at 600 nm (OD_600_) over three consecutive sub-culturing cycles. Strains exhibiting consistently better growth in ADF medium than in DF medium were considered positive for ACC deaminase activity.

### 2.5. Molecular identification and phylogenetic analysis of the acetoin-producing strains

Genomic DNA from selected acetoin-producing strains was extracted. The 16S rRNA gene was amplified via PCR using the universal bacterial primers 27F (5′-AGAGTTTGATCCTGGCTCAG-3′) and 1492R (5′-TACGGCTACCTTGTTACGACTT-3′). The 20 µL PCR mixture contained 2 µL of template DNA, 2 µL of each primer (10 µM), 10 µL of 2× Rapid Taq Master Mix (Vazyme), and 4 µL of ddH_2_O. The amplification products were verified by 1% agarose gel electrophoresis, purified, and sequenced commercially. The obtained sequences were analyzed using the BLAST algorithm, and closely related sequences were used to construct a phylogenetic tree via the neighbor-joining method in MEGA 5.2 software.

### 2.6. Assessment of environmental stress tolerance

Drought stress was simulated using polyethylene glycol 6000 (PEG6000). Seed cultures of the strains were inoculated into GP medium (5 g·L^−1^ glucose, 5 g·L^−1^ K_2_HPO_4_, 7 g·L^−1^ tryptone) containing 0, 5, 10, 20, 30, or 40% (w/v) PEG6000. Growth (OD_600_) was monitored at 12 h intervals for 72 h. Acetoin production was quantified in the 72-h cultures.

For salinity and pH tolerance tests, seed cultures were inoculated into GP medium adjusted to different NaCl concentrations (0 to 2.8 mol·L^−1^) and different pH levels (5.0 to 11.0). Growth (OD_600_) was monitored as described above, and acetoin yield was determined after 72 h of incubation.

### 2.7. Pot experiment with maize seedlings

#### 2.7.1. Preparation of bacterial inoculum, seed sterilization, and pot setup

Selected strains were cultured in modified high salt LB medium (10 g·L^−1^ tryptone, 5 g·L^−1^ yeast extract, 45.1 g·L^−1^ NaCl) at 30 °C, 200 rpm. Cells were harvested by centrifugation at 3700 × g for 10 min, washed, and resuspended in 1/10 Hoagland solution to an OD_600_ of 1.0 for use as the inoculant suspension [23]. Maize seeds were surface-sterilized by rinsing with distilled water, followed by immersion in 75% (v/v) ethanol for 30 sec and 2% (w/v) sodium hypochlorite for 5 min, and finally rinsed thoroughly with sterile water. The sterilized seeds were sown in paper pots with drainage holes containing 320 g of air-dried saline soil. Five seeds were sown per pot. After one week, seedlings were thinned to four uniform plants per pot.

#### 2.7.2. Inoculation, plant growth conditions, and measurement of growth parameters

For the treatment groups, 15 mL of the bacterial suspension (OD_600_ = 1.0) was applied to the rhizosphere of each pot immediately after thinning. Control plants received 15 mL of 1/10 Hoagland solution. A second and third application of 5 mL of suspension (or 1/10 Hoagland solution for controls) was performed at one-week intervals. Each treatment had five biological replicates. The plants were grown in a greenhouse at 25 ± 3 °C, 60%–70% relative humidity, with a photoperiod of 14 h·d^−1^. The seedlings were harvested 50 days after sowing. At the end of the experiment, the following parameters were measured: Plant height, stem diameter, leaf length and width, and fresh and dry weights of shoots and roots. Dry weights were determined after drying the samples at 65 °C to a constant weight.

### 2.8. Statistical analysis

All experiments were performed with at least three independent replicates. Data are presented as mean ± standard error. Statistical significance among treatments was determined by one-way analysis of variance followed by Duncan's multiple range test (*p* < 0.05) using IBM SPSS Statistics 26.0. Figures were generated using Origin 2021 software.

## 3. Results

### 3.1. Screening of acetoin-producing strains and of acc deaminase activity strains

Following isolation and purification, a total of 57 bacterial strains were obtained and designated HPZ-1 to HPZ-57. Screening for specific plant growth-promoting traits identified four strains (HPZ‑43, HPZ‑45, HPZ‑46, and HPZ‑57) that tested positive for ACC deaminase activity. These ACC deaminase-positive strains were reserved for future study. Three strains (HPZ‑9, HPZ‑47, and HPZ‑50) were confirmed as acetoin producers.

### 3.2. Quantification and identification of acetoin‑producing strains

Preliminary screening using the VP test identified 14 VP‑positive isolates among the 57 strains (Table 1). Subsequent quantitative analysis confirmed that three of these strains, HPZ‑9, HPZ‑47, and HPZ‑50, produced significant amounts of acetoin in GP broth, with average yields of 2.30, 2.73, and 2.77 g·L^−1^, respectively.

**Figure 1.**
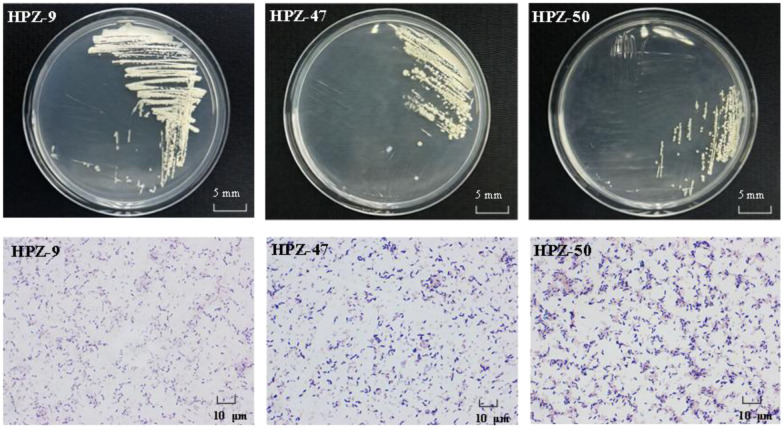
Colony morphology and gram staining of acetoin-producing strains.

Colony morphology observations revealed that HPZ-9 exhibited creamy white, circular, slightly raised, moist, and sticky colonies with relatively regular edges. Strains HPZ-47 and HPZ-50 formed pale yellow, circular, semi-transparent, moist, and viscous colonies. All three strains were Gram-positive short rods (Figure 1).

**Figure 2.**
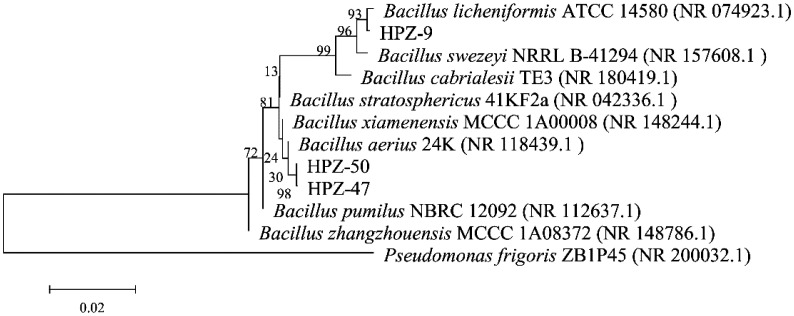
Phylogenetic analysis of acetoin-producing strains.

Phylogenetic analysis based on 16S rRNA gene sequences (Figure 2) classified all three acetoin-producing strains within the genus *Bacillus*. Sequence alignment indicated that strain HPZ‑9 shared 96% similarity with *B. licheniformis*, while strains HPZ‑47 and HPZ‑50 shared 98% similarity with *B. pumilus* and clustered closely on the phylogenetic tree, suggesting a close evolutionary relationship.

**Table 1.** Phenotypic characteristics and acetoin production of *Bacillus* strains.

Strains	ACCD	Gram stain	Colony morphology	VP test	Acetoin production (g·L^−1^)
HPZ-2	–	G^+^	Circular, dry, flat	++	ND
HPZ-6	–	G^+^	Irregular, moist, convex	++	ND
HPZ-7	–	G^+^	Circular, dry, flat	++	ND
HPZ-9	–	G^+^	Circular, dry, convex	++++	2.30
HPZ-19	–	G^-^	Circular, moist, convex	++	ND
HPZ-36	–	G^+^	Circular, dry, flat	++	ND
HPZ-39	–	G^+^	Circular, moist, convex	++	ND
HPZ-41	–	G^+^	Circular, moist, convex	++	ND
HPZ-46	+	G^+^	Circular, dry, convex	+	ND
HPZ-47	–	G^+^	Circular, moist, convex	+	2.73
HPZ-49	–	G^+^	Circular, moist, convex	+	ND
HPZ-50	–	G^+^	Circular, dry, flat	++++	2.77
HPZ-55	+	G^+^	Circular, dry, flat	++	ND
HPZ-57	+	G^+^	Circular, moist, convex	+	ND

*Note: ACCD: ACC Deaminase activity (positive/negative), G⁺/G⁻: Gram-positive/Gram-negative; VP Test: Voges-Proskauer test (number of “+” indicates reaction intensity; “++++” denotes a strong positive result); ND: Not Detected (no measurable acetoin production).

### 3.3. Environmental stress tolerance of acetoin-producing strains

**Figure 3.**
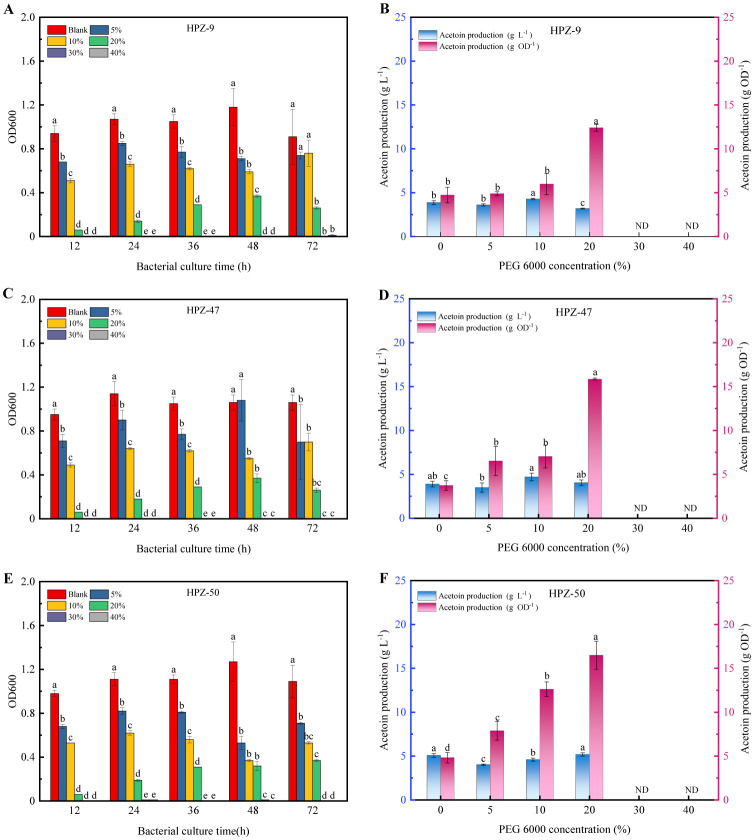
Growth dynamics and acetoin production of *Bacillus* sp. Strains HPZ-9, HPZ-47, and HPZ-50 under PEG6000-induced drought stress. (A, C, E) Growth curves of strains HPZ-9, HPZ-47, and HPZ-50, respectively, monitored 72 h in media containing 0–40% (w/v) PEG6000. (B, D, F) Acetoin production of strains HPZ-9, HPZ-47, and HPZ-50, respectively, after 72 h of incubation under corresponding PEG concentrations. Values are presented as mean ± standard error (n = 3). Different letters above the bars indicate significant differences among treatments according to Duncan's multiple range test (*p* < 0.05).

Under drought stress simulated with PEG6000, bacterial growth was progressively inhibited as PEG concentration increased from 0 to 40% (Figure 3). Growth was nearly completely suppressed at PEG concentrations of 30–40%. Within the 0–20% PEG range, all strains retained the ability to produce acetoin. Yields were 3.19–4.28, 3.50–4.70, and 4.02–5.19 g·L^−1^ for HPZ‑9, HPZ‑47, and HPZ‑50, respectively. Strains HPZ‑9 and HPZ‑47 reached peak acetoin production at 10% PEG, while HPZ‑50 peaked at 20% PEG. Notably, under 20% PEG-induced drought stress, the acetoin production yield normalized by cell growth (acetoin g per OD_600_ unit) increased significantly, averaging 12.4, 15.86, and 16.51 g OD^−1^ for HPZ‑9, HPZ‑47, and HPZ‑50, respectively. This suggests that mild osmotic stress may enhance the specific acetoin synthesis capacity of these strains.

**Figure 4.**
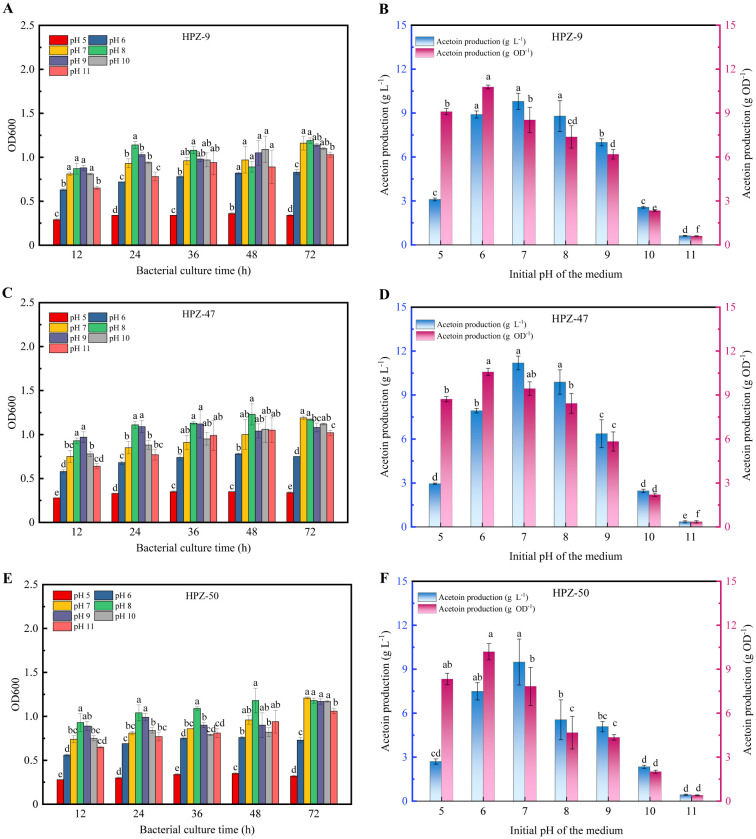
Growth dynamics and acetoin production of *Bacillus* sp. strains HPZ-9, HPZ-47, and HPZ-50 under varying pH conditions. (A, C, E) Growth curves of strains HPZ-9, HPZ-47, and HPZ-50, respectively, monitored for 72 h in media with pH values ranging from 5.0 to 11.0. (B, D, F) Acetoin production of strains HPZ-9, HPZ-47, and HPZ-50, respectively, after 72 h of incubation at corresponding pH levels. Values are presented as mean ± standard error (n = 3). Different letters above the bars indicate significant differences among treatments according to Duncan's multiple range test (p < 0.05).

Strongly acidic conditions (pH 5.0) significantly inhibited the growth of all strains, whereas robust growth was observed under neutral to weakly alkaline conditions (pH 7.0–9.0) (Figure 4). Despite growth variations, all strains produced acetoin across the tested pH range (5.0–11.0), with yields of 0.62–9.8, 0.35–11.19, and 0.43–9.49 g·L^−1^ for HPZ‑9, HPZ‑47, and HPZ‑50, respectively. Maximum acetoin production for all strains occurred at pH 7.0. Even under the inhibitory acidic condition (pH 5.0), acetoin production per unit of cell density remained substantial, averaging 9.94, 9.65, and 9.26 g OD_600_^−1^ for the three strains. Their optimal growth and acetoin production at pH 7–9 align with the alkaline nature of saline-alkali soils. Notably, acetoin synthesis was sustained even when growth was slowed under acidic stress.

**Figure 5.**
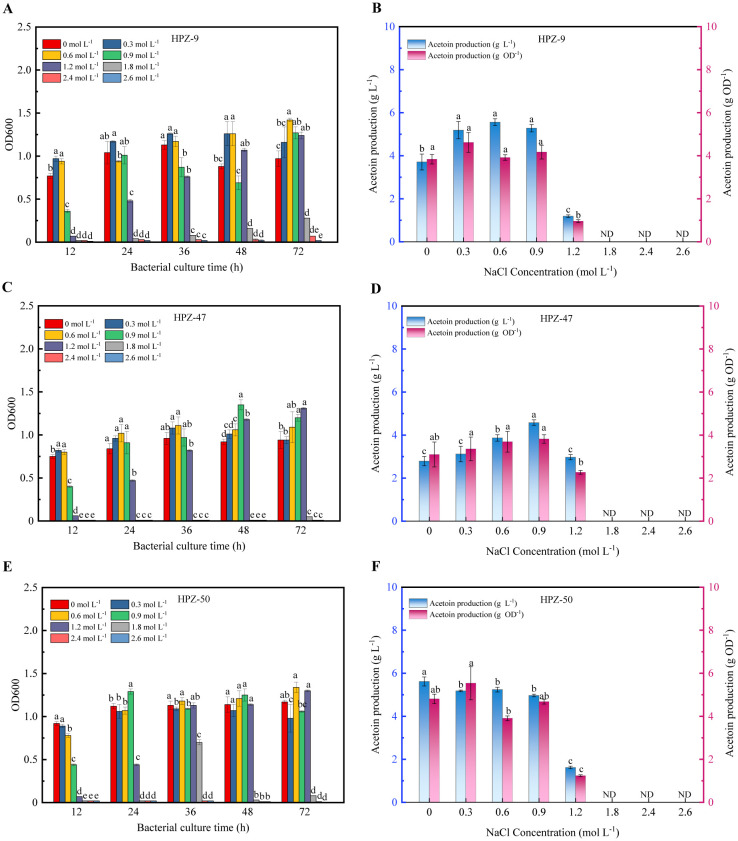
Growth dynamics and acetoin production of *Bacillus* sp. strains HPZ-9, HPZ-47, and HPZ-50 under varying NaCl concentrations. (A, C, E) Growth curves of strains HPZ-9, HPZ-47, and HPZ-50, respectively, monitored for 72 h in media containing 0–2.8 mol L^−1^ NaCl. (B, D, F) Acetoin production of strains HPZ-9, HPZ-47, and HPZ-50, respectively, after 72 h of incubation under corresponding NaCl concentrations. Values are presented as mean ± standard error (n = 3). Different letters above the bars indicate significant differences among treatments according to Duncan's multiple range test (*p* < 0.05).

The optimal NaCl concentration for the growth of all strains was 0.3–1.2 mol·L^−1^ (Figure 5). Growth was significantly inhibited at NaCl concentrations ≥1.8 mol·L^−1^; however, strain HPZ‑9 uniquely demonstrated measurable growth at 2.4 mol·L^−1^ NaCl. All strains produced acetoin under saline conditions, with yields ranging from 1.19–5.56, 2.79–4.58, and 1.62–5.62 g·L^−1^ for HPZ‑9, HPZ‑47, and HPZ‑50, respectively. HPZ‑9 showed higher average acetoin production (5.34 g·L^−1^) within the 0.3–0.9 mol·L^−1^ NaCl range. HPZ‑47 reached its maximum yield (4.58 g·L^−1^) at 0.9 mol·L^−1^ NaCl, while HPZ‑50 achieved its highest yield (5.62 g·L^−1^) in the absence of NaCl. Critically, all three strains maintained robust growth and acetoin production at salinity levels (0.3–1.2 mol·L^−1^ NaCl) typical of saline-alkali soils, with HPZ‑9 exhibiting exceptional tolerance to near-seawater salinity.

### 3.4. Growth-promoting effects on maize seedlings

**Figure 6.**
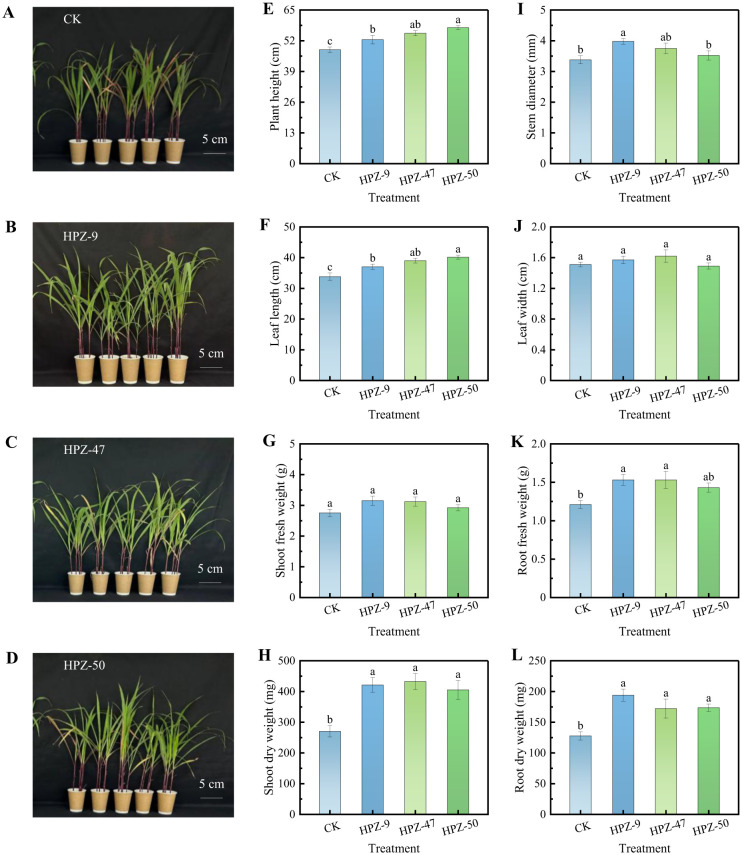
Growth-promoting effects of *Bacillus* sp. strains HPZ-9, HPZ-47, and HPZ-50 on maize seedlings under saline soil conditions. (A–D) Representative images of maize seedlings after 50 days of growth: (A) Non‑inoculated control (CK), (B) inoculated with strain HPZ‑9, (C) HPZ‑47, and (D) HPZ‑50. (E–L) Quantitative effects of bacterial inoculation on morphological and biomass parameters: (E) Plant height, (F) stem diameter, (G) leaf length, (H) leaf width, (I) shoot fresh weight, (J) root fresh weight, (K) shoot dry weight, and (L) root dry weight. Values are presented as mean ± standard error (n = 5). Different letters above the bars indicate significant differences among treatments according to Duncan's multiple range test (*p* < 0.05).

Pot experiment results demonstrated that inoculation with the acetoin‑producing strains HPZ‑9, HPZ‑47, and HPZ‑50 significantly enhanced plant height, leaf length, and shoot and root dry weights of maize seedlings compared to the non‑inoculated control (*p* < 0.05; Figure 6). The most pronounced effects were observed in dry matter accumulation. Shoot dry weight increased by 55.83%, 60.01%, and 49.87% upon inoculation with HPZ‑9, HPZ‑47, and HPZ‑50, respectively. Similarly, root dry weight increased by 51.92%, 34.77%, and 35.94%.

## 4. Discussion

In this study, three acetoin‑producing bacterial strains, HPZ‑9, HPZ‑47, and HPZ‑50, were successfully isolated from the rhizosphere of the halophyte *S. europaea* and preliminarily identified as members of the genus *Bacillus* based on 16S rRNA gene sequencing. Acetoin is an important physiological metabolite excreted by many microorganisms. The excretion of acetoin can be diagnosed by the Voges-Proskauer test and serves as a microbial classification marker [20]. While researchers studying acetoin production by *Bacillus* species have primarily focused on engineered strains or optimized fermentation processes, achieving yields as high as 29.65 to 64.2 g·L^−1^ [24]–[27], the acetoin titers of the wild‑type strains isolated here (2.30–2.77 g·L^−1^ under tested conditions) remain notable. These native strains, isolated from a stressful saline‑alkaline niche, present a valuable genetic resource. Their inherent production capability, coupled with their robust stress tolerance, suggests significant potential for yield enhancement through future metabolic engineering and fermentation optimization aimed at developing effective microbial inoculants.

A key finding of this study is the remarkable environmental adaptability of these strains. They maintained growth and acetoin production under simulated drought (up to 20% PEG6000), a broad pH range (5.0–11.0), and elevated salinity (up to 1.2 mol·L^−1^ NaCl). Notably, mild drought stress (20% PEG) significantly stimulated the specific acetoin production (g per OD unit) in all three strains. This aligns with the concept of acetoin acting as a compatible solute or metabolic regulator in bacterial osmotic‑stress responses [28],[29]. The induction of the acetoin biosynthesis pathway under such conditions may represent a key physiological adaptation, enhancing cellular protection and offering a mechanism by which these PGPR could help plants cope with drought.

The strains exhibited optimal growth and acetoin synthesis at neutral to weakly alkaline pH (7.0–9.0), conditions typical of saline‑alkali soils, which supports their suitability for application in such environments. Importantly, they sustained acetoin synthesis even under growth‑inhibiting acidic stress (pH 5.0), with HPZ‑9 maintaining a high yield per cell unit. This pH resilience, potentially linked to mechanisms like enhanced biofilm formation as seen in other *Bacillus* strains under stress [30], is crucial for rhizospheric colonization and function in fluctuating soil environments.

The three strains grew and produced acetoin at salt concentrations (0.3–1.2 mol·L^−1^ NaCl) relevant to saline soils, with HPZ‑9 demonstrating exceptional tolerance up to 2.4 mol·L^−1^ NaCl. This high salt tolerance is consistent with their isolation source and surpasses the reported salinity limit (0.7 mol·L^−1^ NaCl) for other plant‑growth‑promoting *Bacillus* strains whose acetoin production was not characterized [31],[32]. Interestingly, the strains displayed distinct acetoin production patterns under salt stress: HPZ‑50 produced the most acetoin in the absence of NaCl, while HPZ‑9 maintained high and stable yields across a wider salinity range (0.3–0.9 mol·L^−1^). This functional diversity suggests different metabolic strategies for coping with osmotic stress and provides a rationale for developing tailored or consortia‑based inoculants for soils with varying salinity levels.

The pot experiment confirmed the strong plant growth‑promoting capacity of the isolated *Bacillus* strains in saline soil. The significant enhancement across all key morphological and biomass parameters, particularly the marked increase in dry matter accumulation over fresh weight (Figure 6), suggests that inoculation not only stimulates growth but also improves plant water‑use efficiency or carbon partitioning under stress. This aligns with the documented role of bacterial volatiles like acetoin in modulating plant physiology under adverse conditions. For instance, exogenous acetoin has been shown to upregulate photosynthesis and antioxidant defense in lettuce under salinity, indicating a systemic priming effect [33]. The performance of our strains, especially in promoting structural biomass, compares favorably with reports on other *Bacillus* inoculants, such as *B. subtilis* NRCB002 [34], underscoring their efficacy even as wild‑type strains. Notably, the observed phenotypic variation among the strains, where HPZ‑50 excelled in promoting vertical growth, while HPZ‑9 and HPZ‑47 were more effective in enhancing shoot and root dry weight (Figure 6), suggests distinct functional emphases. This phenotypic variation implies potentially distinct mechanistic emphases among the strains, which could be leveraged in consortium formulations.

Beyond their efficacy, the ecological origin and stress-adapted physiology of HPZ-9, HPZ-47, and HPZ-50 make them particularly promising for saline-alkali soil remediation. Among PGPR applied to such challenging environments, *Bacillus* species often demonstrate superior performance in improving crop yield and quality compared to genera like *Pseudomonas* [35],[36]. This study adds to this repertoire by providing three novel, acetoin‑producing *Bacillus* strains endowed with exceptional environmental resilience. Their ability to maintain growth and metabolite production under a combination of osmotic, pH, and ionic stresses aligns with the multifactorial nature of field conditions. This functional versatility offers more diversified and resilient solutions for microbial‑assisted remediation of degraded saline‑alkali lands and for enhancing agricultural productivity under marginal conditions.

## 5. Conclusions

Three novel acetoin‑producing *Bacillus* strains (HPZ‑9, HPZ‑47, HPZ‑50) were isolated from the rhizosphere of the halophyte *S. europaea*. They exhibited broad tolerance to pH (5–11), salinity (0.3–1.2 mol·L^−1^ NaCl), and osmotic stress (20% PEG6000). In pot experiments, these strains significantly enhanced maize seedling biomass under saline conditions, demonstrating their potential as PGPR for improving crop resilience in saline‑alkali soils. In future work, researchers should focus on genome sequencing, field validation, and elucidating the mechanism of acetoin‑mediated growth promotion.

## Use of AI tools declaration

The authors utilized AI-assisted editing tools (DeepSeek) solely for the purpose of improving language fluency and readability. The final content was reviewed and verified by the authors.
